# Validation of the brief Adjustment Disorder New Modules with Australian oncology patients

**DOI:** 10.1186/s13030-022-00259-w

**Published:** 2023-01-25

**Authors:** Bernadette E. Harris, Kylie Rice, Clara V. Murray, Einar B. Thorsteinsson

**Affiliations:** School of Psychology, University of New England, Armidale, NSW 2351 Australia

**Keywords:** Adjustment disorder, ADNM-8, ADNM-4, Validation, Oncology, Psycho-oncology

## Abstract

**Background:**

Evidence suggests that up to 30% of cancer patients may meet the criteria for adjustment disorder. However, no assessment instruments have been validated for use with cancer patients. The Adjustment Disorder New Module (ADNM)-8 and ADNM-4 are brief screening tools for adjustment disorder mapped directly to the new ICD-11 criteria. The aim of this study was to investigate the factor structure and validity of both instruments in an Australian sample of adult oncology patients.

**Methods:**

A total of 405 participants with a cancer diagnosis were recruited online from across Australia. Participants reported cancer-specific information, such as time since diagnosis, treatment stage, cancer stage, type of cancer, and the following questionnaires: 8-item Adjustment Disorder New Module (ADNM-8), the World Health Organisation Well-Being Index (WHO-5), and the short form Depression Anxiety and Stress Scale (DASS-21). The predictiveness of stressors was assessed using multiple regression analysis and the structure of the ADNM-8 and the ADNM-4 was tested using confirmatory factor analysis.

**Results:**

Six previously tested models were examined, and the results suggested a 2-factor structure reflecting the two ICD-11 diagnostic criteria clusters of preoccupation with the stressor and failure to adapt was a good fit for both scales. The ADNM-4 outperformed the longer version of the scale on numerous fit indices though the ADNM-8 and ADNM-4 were highly correlated. Correlations of both scales with the psychological distress scale, the stress subscale, and the wellbeing index indicated good construct validity.

**Conclusions:**

Our results suggest that the ADNM-8 and ADNM-4 are useful screening tools for assessing adjustment disorder symptoms in cancer patients. The prompt screening of cancer patients encourages early intervention for those at risk of adaptation difficulties and informs research and clinical decisions regarding appropriate treatments.

**Supplementary Information:**

The online version contains supplementary material available at 10.1186/s13030-022-00259-w.

## Background

In Australia, cancer causes extensive morbidity and mortality [1]. Estimates suggest that in 2021 there will be 151,000 new cancer diagnoses and 49,000 cancer-related deaths [2]. The incidence of cancer is growing relative to the ageing population, and the risk of cancer increases markedly from the age of 35 [2, 3]. However, survival rates are increasing due to improved screening and treatments, increasing the need to understand the practical and psychological adjustment needs and experiences of those in survivorship [2]. Cancer places a huge social and economic stress on individuals, families, and the community. Increased incidence and survival rates have implications for health and welfare service provision, including the need for sensitive screening instruments and appropriate psychological interventions which facilitate healthy psychological adjustment and, in turn, optimise quality of life for those living with cancer [4].

### Adjustment disorder

In 2018 the World Health Organization (WHO; [5]) released a new definition of adjustment disorder (AjD): a maladaptive response to a stressor (e.g., a cancer diagnosis, family or work conflicts) that appears within a month of the stressor. The International Classification of Diseases (ICD-11; [5]) identifies AjD as having the following differentiating features, 1) a preoccupation with, and 2) a failure to adapt to the stressful event ([5], 6B43). The improved clarity offered by the new definition suggest it is timely to investigate the properties of screening instruments that map to the new diagnostic criteria.

AjD is associated with significant functional impairment and reduced quality of life [6], and an increased relational risk [7]. If left untreated, AjD may increase suicide risk [8, 9] and heighten suicidal behaviour [10]. A longitudinal study of injury survivors by O’Donnell et al. [11] found that the presence of AjD at 3-months post-injury increased the likelihood of another more severe psychiatric disorder (e.g., major depressive disorder, posttraumatic stress disorder) at 12-months. If this is correct, early detection of adjustment issues is critical, and prompt intervention may disrupt this prognosis [11]. In the cohort of injury survivors, O'Donnell et al. found that AjD was a chronic condition in one-third of their sample, suggesting that AjD may be a pervasive disorder in some people. Given the nature of cancer and the impact of cancer treatments (e.g., pain, fatigue), and because the effects of cancer can be long term, it seems at least possible that for some oncological patients, AjD may be a persistent rather than transient disorder. Despite having been cast as a less severe disorder, and labelled the “wastebasket” of diagnoses [12], it is associated with considerable individual and relationship distress, reduction in quality of life, decreased functionality (e.g., sleep disturbances), increased suicidal ideation [13] and risk of death by suicide [14], and as such warrants greater attention in research.

### AjD and cancer

AjD commonly presents in the context of a cancer diagnosis; however, there is sparse extant research on the disorder in this context [12, 15]. A meta-analysis of 23 studies in oncological and haematological settings found that the prevalence of AjD in cancer patients was 19.4% and in palliative care settings it was 15.4% [16]. An Australian longitudinal study of people who experienced AjD after major injury assessed prevalence at a similar level [11]. Horn [17] found prevalence estimates of AjD at similar levels in patients experiencing multimorbidity (including cancer patients). Recently Van Beek et al. [18] assessed the prevalence of AjD in cancer patients after treatment as between 13–15%. Given the preponderance of assessments cancer patients undergo, any psychological assessments must be valid, reliable, and brief to have any clinical utility. In addition to being efficient, brief instruments can also reduce the erroneous comorbidity of disorders when they focus on the core elements of a disorder [19, 20]. There are only two brief AjD specific screening tools available to clinicians; those based on the Adjustment Disorder New Module (ADNM; [21]) and the International Adjustment Disorder Questionnaire [22], which is longer than both versions of the ADNM examined in this study. Most structured clinical interviews do not include assessment of the disorder, and those that do usually have very few items and only if the threshold for other disorders is unmet [23]. Given the prevalence, and possible impairment, related to a cancer diagnosis, research into AjD in this population is greatly needed.

### AjD assessment

Kazlauskas E et al. [6] investigated the psychometric properties of a shortened 8-item scale (ADNM-8) with a sample of individuals who registered for a stress management program. The 8-item scale included the list of stressors and the core AjD symptoms (preoccupation and failure to adapt). Kazlauskas et al. found the 8-item scale to have two factors aligned with the two core symptom groups, with good construct validity and internal reliability. Later Ben-Ezra et al. [19] examined the construct validity of the ADNM-8 with a representative Israeli national sample and extended the Kazlauskas study by testing a new Ultra-Brief 4-item scale (ADNM-4). In addition, they established cut-off scores for each scale. Ben-Ezra’s study found that a 7-item (the ADNM-8 less the functional impairment item) two-factor model was a better fit than an AjD one-factor model. Concerning the ADNM-4, the two-factor model outperformed the unidimensional structure tested [19]. The Ben-Ezra et al. [19] study showed evidence for the diagnostic usefulness of each scale, particularly their ability to rule out AjD. In 2019, Lavenda et al. [24] conducted a revalidation of the ADNM-4 with a non-clinical sample of Israelis adults. They found that a unidimensional factor fit the data well using exploratory factor analysis and confirmatory factor analysis (CFA). The validity of these brief scales appears to be supported. However, there is some discrepancy regarding the factor structure of the ADNM-4 (i.e., whether there is a unidimensional or bidimensional factor structure and if it is the same for both scales). In addition to recommending the factor structure of the scales be clarified, prior research urges future studies focus on specific stressor groups, targetted populations, and other cultures to check the psychometric properties of the instruments further [6, 19, 21, 24]. Importantly, examining the psychometric properties with different cultures and targetted populations establishes the degree of clinical utility and appropriateness of the scales for use within that culture and with that particular population.

### The present study

Researchers have not yet assessed the psychometric properties of the ADNM-8 or ADNM-4 with an oncological sample or an Australian sample. As such, the present study aims to test the factor structure of the English versions of the ADNM-8 and ADNM-4 scales and to assess which of the factor structures identified in previous validation studies i.e., [6, 19, 24] hold for an Australian sample of adults coping with the effects of cancer. The investigation includes examining the psychometric properties of the English versions of the ADNM-8 and ADNM-4 in an adult sample of Australian cancer patients. The present study will replicate and extend the original validation study by Kazlauskas et al. [6] and replicate the AjD sections of the study by Ben-Ezra et al. [19]. Furthermore, this study will address the gaps in the study by Kazlauskas et al. by assessing both convergent and discriminant validity. CFA will test the scales' hypothesised structure, the prevalence of stressors will be investigated, and the predictive utility of stressors will be examined using multiple regression. Therefore, the following hypotheses are proposed:

- H1 : That AjD scores will be positively associated with psychological distress and negatively associated with wellbeing.
- H2: Given previous research, the cohort, and timing of the study, the researchers considered that the participants’ own serious illness and the impact of COVID-19 would be the most commonly reported stressors.
- H3: That there would be gender effects in the prevalence of stressors with women reporting death and illness of a loved one, moving, and family conflicts more often than men,
- H4: That the strongest predictor of AjD scores would be participants’ own serious illness.
- H5: That participants would report cancer and its consequences as the most straining stressor.
- H6: Given the findings of O’Donnell et al.[11], the authors’ proposed that AjD scores would be equivalent for those diagnosed within the past 12 months compared with those diagnosed longer than 12 months.
- H7: Finally, the researchers hypothesised that the ADNM-8 and ADNM-4 would reflect the ICD-11 criteria and show a bifactor structure.

## Method

### Design

This study uses a cross-sectional design to examine relationships between item responses (indicators) and latent variables (factors) in the ADNM-8 and ADNM-4 screening scales.

### Participants

Following a university ethics committee approval, the research team advertised the study on social media between September 2020 and April 2021. Advertising invited people residing in Australia over 18 and diagnosed with cancer to participate in the study. Recruitment resulted in 405 participants who completed the ADNM-8 scale, the validity scales, and demographic information as part of a larger battery of measures. The age range of participants was 35 to 85 years (*M* = 59.73, *SD* = 8.73), with most participants being female (74%), heterosexual (95%), and married (67%). Most participants identified as from an Australian cultural background (78%), with Aboriginal and Torres Strait Islanders represented similarly to their representation in the same-age Australian general population (2%). Participants came from all over Australia, with a slight majority from rural and remote areas (56%).

All participants had a cancer diagnosis; most females reported breast cancer (61%), followed by ovarian cancer (8%), whereas most males were diagnosed with prostate cancer (32%), followed by bowel cancer (16%). Table 1 shows the full list of cancer types in the sample. Many participants reported having Stage 3 or 4 cancer (40.5%), and similar numbers reported being in the treatment or treatment review stage (35%). Table 2 provides the breakdown of cancer and treatment stages of those in the sample. Time since diagnosis was on average 4.6 years (*SD* = 6.12), although 29% of the sample had been diagnosed within the past 12 months. Many patients travelled significant distances to see their oncologist (*M* = 65.29 km, Range = 0–1600 km, *SD* = 148.8 km), some travelling more than 120 kms (14%).

**Table 1:** Types of cancers in the sample (*N* = 405)

Cancer type	*N*	%
Breast cancer	185	45.7
Prostate cancer	34	8.4
Bowel cancer	30	7.4
Ovarian cancer	24	5.9
Melanoma	15	3.7
Non-Hodgkin lymphoma	15	3.7
Lung cancer	11	2.7
Bladder cancer	10	2.5
Blood cancers	8	2.0
Uterine cancer	7	1.7
Bone and Bone Marrow cancers	7	1.7
Kidney cancer	6	1.5
Hodgkin lymphoma	5	1.2
Cervical cancer	5	1.2
Keratinocyte cancers	5	1.2
Throat cancer	4	1.0
Soft tissue sarcoma	4	1.0
Other cancers	30	7.4

**Table 2:** Cancer and treatment stage of participants in the sample (*N* = 405)

Variable	*N*	%
Cancer stage		
Stage 0	31	7.7
Stage 1	57	14.1
Stage 2	85	21.0
Stage 3	88	21.7
Stage 4	76	18.8
I don’t know	68	16.8
Treatment stage		
Pre-treatment planning	9	2.2
Treatment	116	28.6
Treatment review	26	6.4
Post-treatment monitoring	155	38.3
Remission review	59	14.6
Palliative care	13	3.2
Other, please specify	27	6.7

### Measures

#### Demographic information

The online questionnaire collected patients’ home postcode, age, sex assigned at birth, current gender, sexuality, relationship status, length of time in the relationship, number of children cared for, cultural background, and number of people residing at the home address.

#### Cancer-specific information

Participants reported the length of time since their cancer diagnosis, their treatment stage, cancer stage, type of cancer, and the number of kilometres they travel to their oncologist.

#### Adjustment disorder

The 8-item Adjustment Disorder New Module [6] is a brief screening tool that assesses ICD-11 symptoms of AjD. The scale includes two components 1) a list of stressors and 2) AjD symptom criteria. The stressor list comprises acute and chronic stressors and asks participants to select all that have impacted them in the past two years. One stressor was added to the list to reflect the current pandemic, 'The impact of COVID-19'. Participants then identify the most straining events from those previously selected. The second section has eight items—two 4-item subscales that address the core ICD-11 AjD symptoms: preoccupation (e.g., “I have to think about the stressful situation a lot and this is a great burden to me”) and failure to adapt (e.g., “Since the stressful situation, I find it difficult to concentrate on certain things”). The ADNM-8 measures each item on a 4-point scale from 1 (*Never*) to 4 (*Often*). Each subscale item is added to produce subscale scores, and summing all items provides a total score. Participants indicate how long they have experienced this reaction indicating if less than 1-month, 1-month to 6-months, or 6-months to 2-years. The ADNM-8 has previously shown adequate to good internal reliability with Cronbach’s alphas for the subscales of Preoccupation α = 0.85 and Failure to Adapt α = 0.71, and the total ADNM-8 scale α = 0.83 [6]. The ADNM-4 is a very brief version of the ADNM-8 with only 4 questions, two from each subscale. The ADNM-4 has shown adequate reliability with α = 0.81 [19]. Table 3 presents the items in both the ADNM-8 and ADNM-4.

**Table 3:** Items on the ADNM-4 and ADNM-8 Scales

Item	Statement	ADNM-4	ADNM-8
1	I have to think about the stressful event repeatedly	-	Included
2	I have to think about the stressful situation a lot and this is a great burden to me	Included	Included
3	Since the stressful situation, I find it difficult to concentrate on certain things	Included	Included
4	I constantly get memories of the stressful situation and can't do anything to stop them	Included	Included
5	My thoughts often revolve around anything related to the stressful situation	-	Included
6	Since the stressful situation, I do not like going to work or carrying out the necessary tasks in everyday life	Included	Included
7	Since the stressful situation, I can no longer sleep properly	-	Included
8	Overall, the stressful situation affected me strongly in my personal relationships, my leisure activities, or in other important areas of life	-	Included

*Note*. Answer anchors for each item = never, rarely, sometimes, often

Ben-Ezra et al. [19] used a ROC analysis to test the ‘theoretical algorithm’ for an ICD-11 AjD diagnosis and proposed a cut-off score of 18.5 for clinical use with the ADNM-8 scale and 8.5 for the ADNM-4. Above these scores, Ben-Ezra et al. found that an AjD diagnosis was highly likely. These cut-off sores are currently recommended for clinical use.

#### Wellbeing

Wellbeing was measured using the 5-item WHO Well-Being Index (WHO-5; [25]), which assesses subjective psychological wellbeing over the past two weeks. The WHO-5 includes positively phrased statements reflecting current wellbeing (e.g., “I have felt cheerful and in good spirits”). The WHO-5 is considered unidimensional, free of diagnostic specificity, and is deemed a clean generic scale for the measure of wellbeing [26]. The WHO-5 has shown sensitivity to treatment change with oncology patients [27], and has been used extensively in stress-related studies, and has reported high clinometric validity [26]. In the present study, the WHO-5's internal consistency was assessed by Cronbach’s alpha 0.89.

#### Psychological distress

The 21-item Depression Anxiety and Stress Scale (DASS-21; [28]) measures the frequency of negative emotional states during the past week. The DASS-21 has three 7-item subscales: depression, anxiety, and stress. These subscales combine to measure a more general dimension of psychological distress [29], with higher scores indicating greater levels of distress. The DASS-21 subscales distinguish well between depression, anxiety, and stress [29, 30]. The DASS-21 has been used previously in studies using the Adjustment Disorder New Modules [31], and has been shown to have good psychometric properties in both Australian and oncological samples [29, 32, 33]. Table 4 shows the DASS-21 means and standard deviations for the present study. The DASS-21 has previously demonstrated good internal consistency. In the current study, Cronbach's alphas were depression 0.91, anxiety 0.83, stress 0.88, and 0.94 for the total scale.

**Table 4:** Means, standard deviations, and correlations between the ADNM-8, ADNM-4, DASS-21 subscales and totals, and the WHO-5 (*N* = 405)

Measure	*M*	*SD*	1	2	3	4	5	6	7	8	9	10
1. ADNM-8 Subscale: Preoccupation	11.73	2.96	–									
2. ADNM-8 Subscale: Fail to Adapt	10.56	3.11	.72**	–								
3. ADNM-8 Total	22.29	5.62	.92**	.93**	–							
4. ADNM-4 Subscale: Preoccupation	5.73	1.56	.96**	.68**	.88**	–						
5. ADNM-4 Subscale: Fail to Adapt	5.00	1.67	.69**	.91**	.86**	.66**	–					
6. ADNM-4 Total	10.72	2.96	.90**	.88**	.96**	.90**	.92**	–				
7. DASS-21 Subscale: Depression	6.15	4.55	.42**	.51**	.50**	.42**	.50**	.51**	–			
8. DASS-21 Subscale: Anxiety	4.63	3.87	.40**	.47**	.47**	.41**	.46**	.48**	.66**	–		
9. DASS-21 Subscale: Stress	7.00	3.92	.46**	.52**	.53**	.46**	.50**	.53**	.70**	.73**	–	
10. DASS-21 Total	17.77	11.03	.48**	.56**	.56**	.48**	.55**	.57**	.89**	.88**	.90**	–
11. WHO-5	47.60	21.70	-.42**	-.53**	-.52**	-.42**	-.48**	-.49**	-.66**	-.51**	-.56**	-.65**

*Note*. Associations tested with Pearson’s *r* with 2000 bootstraps

^**^p < 0.01 (one-tailed)

### Statistical analysis

The predictiveness of stressors was assessed using multiple regression analysis. The structure of the ADNM-8 and the ADNM-4 was tested using confirmatory factor analysis. An a priori power analysis estimated the sample size required for model structure as 100 and 90 to detect an effect size of 0.3 with alpha of 0.05 and 80% power [34]. Tabachnick and Fidell [35] argue that good fitting models perform well on several indices. Therefore, several fit indices are reported to provide an overall picture of the performance of each model. Criteria levels used are those recommended by Tabachnick and Fidell [35] and Medsker et al. [36]. The ADNM-4 analysis was based on responses given to the 8-item scale. We tested six models in total: four ADNM-8 models and two ADNM-4 models. The first two models tested were replicated from the study by Kazlauskas et al. [6]. Model 1 was a 2-factor, 8-item model comprised of two latent factors (preoccupation and failure to adapt) with four items loaded onto each factor, reflecting the core AjD symptoms and one item representing functional impairment [6]. Model 2 comprised Model 1 with the addition of a correlation between Items 1 and 2. Models 3–6 were replicated from the study by Ben-Ezra et al.[19]. Model 3 was a 2-factor, 7-item model comprised of two latent factors (preoccupation and failure to adapt) with four items loaded onto preoccupation and three items onto failure to adapt. Consistent with Ben-Ezra et al.’s [19] second model, the functional impairment item was omitted. The fourth model tested was a one-factor, 7-item model, which included the core AjD symptoms without the functional impairment item, with all items loading onto a single general AjD factor [19]. The fifth and sixth models examined the ultra-brief ADNM-4 scale. Each model comprised two items from each cluster of symptoms: Items 2, 3, 4 and 6 taken from the ADNM-8. Model 5 tested a 2-factor version, and Model 6 examined a general AjD factor. Confirmatory factor analysis with maximum likelihood estimation was calculated with R Version 4.0.5 and RStudio Version 1.4.1106 to examine the ADNM-8 and ADNM-4. All other data analyses used IBM SPSS Statistics Version 27.

## Results

### Descriptive statistics, reliability, and correlations

The mean score for the ADNM-8 was 22.29 (*SD* = 5.62), and the ADNM-4 was 10.72 (*SD* = 2.96) in the current study sample. Table 4 lists descriptive statistics for the sample. Cronbach’s alphas indicated high internal reliability for both the ADNM-8 and ADNM-4. Internal reliability for the ADNM-8 was α = 0.90 and for the Ultra Brief ADNM-4, α = 0.81.

To test the first hypothesis and examine the construct validity of the two scales the relationship between the scales and between both scales and the DASS-21 and the WHO-5 were examined using a Pearson’s product-moment correlation. Gignac and Szodoral [37] recommend revised normative guidelines for interpreting correlations, 0.10, 0.20, and 0.30 as small, medium, and large, respectively. Table 4 includes the correlations tested. Correlations between the two versions of the ADNM under consideration, are significant and large, 0.96 meaning the ADNM-4 is capturing the same construct and therefore indicates the ADNM-4 can be used as a replacement for the longer version of the scale. Correlations were significant and large between both ADNM scales, and the DASS-21 total and its subscales. This result points to the convergent validity of the ADNM scales. The ADNM scales also had consistently larger correlations with the depression subscales and stress subscales than with the anxiety subscale. Further, both versions of the ADNM had significant and large negative correlations with the WHO-5 supplying support for discriminant validity.

### Prevalence of stressors by gender

To test the second hypothesis the frequency of stressors was assessed. Participants had experienced an average of 4.07 (SD = 2.13) stressors in the two years preceding the survey, ranging from one to thirteen stressors. Most of the sample (89.4%) experienced multiple life stressors. The prevalence of stressors appears in Table 5. The most selected stressors were own serious illness and the impact of COVID-19. In testing the third hypothesis we found significant gender effects for several stressors. Compared with men, women reported family conflict, the impact of COVID-19, and the death of a loved one more frequently as significant life stressors. Table 5 provides the detail of stressors selected and the gender effects.

**Table 5:** Prevalence of life stressors (*N* = 405)

	Total		Male		Female		Gender effect	Effect size
	(*N* = 405)		(*N* = 105)		(*N* = 300)			
Life stressors	*N*	%	*N*	%	*N*	%	χ^2^	ɸ
The impact of COVID-19	220	54.3	45	42.9	175	58.3	7.51**	0.14
Acute stressors								
Death of a loved one	103	25.4	18	17.1	85	28.3	5.14*	0.11
Divorce/separation	24	5.9	5	4.8	19	6.3	0.35	0.03
Moving	58	14.3	15	14.3	43	14.3	0.00	0.00
Assault / Criminal act	6	1.5	2	1.9	4	1.3	0.17	0.02
Retirement	80	19.8	27	25.7	53	17.7	3.18	0.09
Termination of an important leisure activity	77	19.0	16	15.2	61	20.3	1.31	0.06
Serious accident	10	2.5	1	1.0	9	3.0	1.35	0.06
Natural disasters	4	1.0	1	1.0	3	1.0	0.00	0.00
Chronic stressors								
Financial difficulties	132	32.6	37	35.2	95	31.7	0.45	0.03
Family conflict	145	35.8	20	19.0	125	41.7	17.31***	0.21
Own serious illness	268	66.2	63	60.0	205	68.3	2.41	0.08
Conflict at work	79	19.5	14	13.3	65	21.7	3.44	0.09
Conflict with neighbours	37	9.1	10	9.5	27	9.0	0.03	0.01
Too much/ too little work	113	27.9	24	22.9	89	29.7	1.80	0.07
Illness/care of a loved one	145	35.8	31	29.5	114	38.0	2.43	0.08
Unemployment	56	13.8	10	9.5	46	15.3	2.20	0.07
Pressure to meet deadlines	68	16.8	12	11.4	56	18.7	2.92	0.09
Other	22	5.4	6	5.7	16	5.3	0.02	0.01

^*^p < .05, **p < .01, ***p < .001

### Stressor predictors of AjD

A multiple regression analysis examined the predictiveness of stressors to test the fourth hypothesis. Nineteen stressors entered in one block accounted for 25.4% of the variance in ADNM-8 AjD scores, *R*^2^ = 0.25, *F*(19.385) = 6.91, *p* < 0.001. The regression results appear in Table 6. In order of influence, the stressors that explained the most significant amounts of unique variance in ADNM-8 scores were: 1) financial problems (3.28%), 2) own serious illness (3.06%), 3) family conflicts (2.37%), 4) conflicts with neighbours (1.80%), 5) the impact of COVID-19 (1.54%), 6) natural disasters and other stressors (1.35% each), and 7) divorce/separation (1.19%). These eight stressors combined account for 17.11% of the variance in ADNM-8 scores. The same stressors were also the most predictive of both subscale scores; however, the strength of their influence varied: For the preoccupation subscale, the top stressors were family conflicts (2.62%), financial problems (1.93%), and the impact of COVID-19 (1.80%). Own serious illness was the fifth most influential predictor of preoccupation (1.61%). The top stressors for the failure to adapt subscale were own serious illness and financial problems (3.84% each), followed by conflicts with neighbours (1.77%).

**Table 6:** Unstandardised (B) and standardised (*β*) regression coefficients and semi-partial correlations (sr) for life stressors as predictors of ADNM-8 AjD scores (*N* = 405)

Life stressors	*B*	95% CI for *B*		β	*t*	*p*	*sr*
		*LL*	*UL*				
The impact of COVID-19	1.47	0.45	2.50	.13	2.83	.005	.12
Acute stressors							
Death of a loved one	-0.90	-2.07	0.26	-.07	-1.52	.128	-.07
Divorce/separation	2.70	0.56	4.85	.11	2.48	.014	.11
Moving	-0.25	-1.71	1.21	-.02	-0.34	.736	-.02
Assault / Criminal act	0.59	-3.63	4.81	.01	0.28	.784	.01
Retirement	-0.07	-1.34	1.20	-.01	-0.11	.913	-.01
Termination of an important leisure activity	0.14	-1.17	1.45	.01	-0.21	.832	.01
Serious accident	0.69	-2.48	3.85	.02	0.43	.670	.02
Natural disasters	6.81	1.73	11.89	.12	2.64	.009	.12
Chronic stressors							
Financial difficulties	2.35	1.23	3.47	.20	4.12	.001	.18
Family conflict	1.87	0.82	2.93	.16	3.50	.001	.15
Own serious illness	2.15	1.09	3.21	.18	3.98	.001	.18
Conflict at work	0.94	-0.39	2.26	.07	1.39	.165	.06
Conflict with neighbours	2.71	0.96	4.46	.14	3.05	.002	.13
Too much/ Too little work	0.45	-0.79	1.69	.04	0.72	.474	.03
Illness/care of a loved one	0.66	-0.41	1.72	.06	1.21	.225	.05
Unemployment	0.44	-1.04	1.91	.03	0.58	.560	.03
Pressure to meet deadlines	0.32	-1.15	1.78	.02	0.42	.672	.02
Other	2.95	0.76	5.15	.12	2.64	.009	.12

*Note*. *CI* Confidence interval, *LL* lower limit, *UL* upper limit

Given the broad range in the time since diagnosis in the data (from 0.08 to 49 years), secondary analyses were conducted to consider if the length of time since diagnosis influenced the stressors reported. Statistical analysis was completed to examine the stressor profiles for those with a diagnosis of less than one year, five years, seven and a half years, and ten years. These time points were selected based on the positive skew of the sample (3.00). The full details of each analysis are provided in the Supplementary Material. Model results for each regression appear in Table 7.

**Table 7:** Results of multiple regressions of the predictiveness of life stressors on ADNM-8 Scores for different time since diagnosis groups

Time since diagnosis	N	*R*^*2*^	*df*	*F*	*p*	% of total cases
≤ 1 year	118	.35	19, 98	2.72	.001	29
≤ 5 years	296	.28	19, 276	5.62	.001	73
≤ 7.5 years	328	.26	19, 308	5.82	.001	81
≤ 10 years	351	.29	19, 331	7.09	.001	87

*Note. Df* degrees of freedom

The major differences between the timepoints were that for those diagnosed one year or less ‘The impact of COVID-19’ was twice that of any other timepoint (*B* = *3.05*). Divorce or separation had a greater influence on the ADNM-8 score as the time since diagnosis increased, while death of a loved one had a lessor affect. ‘Financial difficulties’ remained constant across all timepoints, while ‘Own Serious Illness’ had a much greater influence on the ADNM-8 scores beyond the one year since diagnosis time point. ‘Family conflict’ stays relatively constant across all time since diagnosis points however it is lowest for those whose diagnosis came one year or less. The influence of ‘Natural disasters’ on the total ADNM-8 score has an inverse relationship to the time since diagnosis with the one or less timeframe reporting the largest effect. Refer to Tables 1, 2, 3 and 4 in the Supplementary Material.

### Most straining events

The fifth hypothesis examined participants’ reports of the most straining events of those stressors they had previously selected. Participants identified cancer and its related effects such as treatment, medication, pain, or immobility as the most straining event experienced (49.4%), followed by illness/death of a loved one (13.1%), relationship issues (10.4%), and COVID-19 related factors (9.9%). Cancer in the context of the pandemic as was nominated as the most straining event experienced by 10.4%, many referring to having to undergo treatment without family support and being unable to visit or have visits from family while undergoing treatment.

### Chronicity of AjD

To assess the sixth hypothesis an independent samples *t*-test compared the average ADNM-8 scores reported by those diagnosed within the last 12 months (*n* = 118) to the average AjD score reported by those whose diagnosis was less recent (*n* = 287). The *t*-test revealed there were no significant differences between these groups on their AjD scores. The recent diagnosis group (*M* = 22.72, *SD* = 0.46) reported scores 0.61 points higher on the ADNM-8, 95% CI [-0.519, 1.74] than the less recent diagnosis group (M = 22.11, SD = 0.35) however there was more variance in the less recently diagnosed cohort, *t*(256.64) = 1.06, *p* = 0.289, two-tailed, Cohen’s *d* = 0.11.

### ADNM-8 and ADNM-4 factor structures

**Fig. 1:**
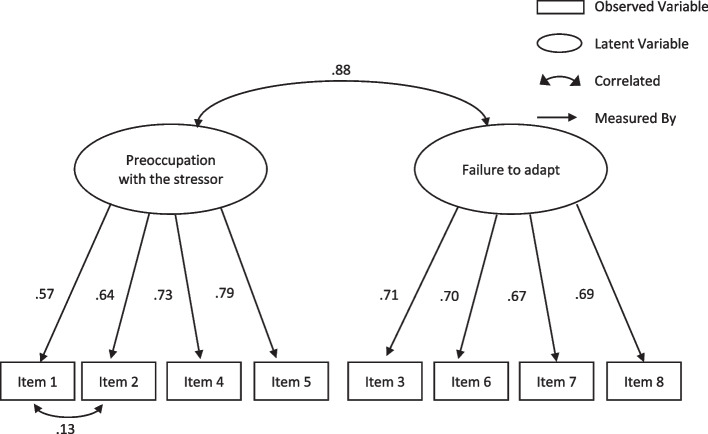
Standardised solution of the 2-factor model of the ADNM-8 referred to as Model 2 in Table 8. Note. All modelled correlations and path coefficients are significant (*p* < .001)

**Fig. 2:**
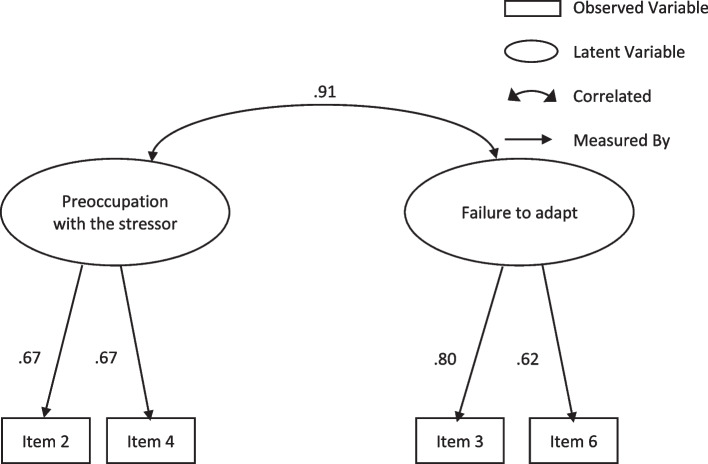
Standardised solution of the 2-factor model of the ADNM-4 referred to as Model 5 in Table 8. Note. All modelled correlations and path coefficients are significant (*p* < .001)

To evaluate the final hypothesis a CFA was conducted on six models, two models previously tested by Kazlauskas et al. [6] and four models previously tested by Ben-Ezra et al. [19]. Table 8 presents the results. Of the four ADNM-8 models tested, the revised Kazlauskas et al.model, shown in Table 8 as Model 2, was the best fit of the 8-item models tested with this sample. The correlation between the two factors and factor loadings for Model 2 appears in Fig. 1. Of the two ADNM-4 models tested, the two-factor model, shown in Table 8 as Model 5, fit this sample better. The correlation between the two factors and factor loadings for Model 5 appear in Fig. 2.

**Table 8:** Summary of fit indices for the six models maximum likelihood—standardised regression weights (*N* = 405)

Fit index	Criteria	Kazlauskas Models		Ben-Ezra Models			
		ADNM-8				ADNM-4	
		Model 1	Model 2	Model 3	Model 4	Model 5	Model 6
χ2		122.210	69.055	92.453	121.803	0.397	6.253
*p*		< 0.001	< 0.001	< 0.001	< 0.001	0.529	0.044
*df*		19	18	13	14	1	2
χ2 /*df*	≤ 2.00	6.43	3.836389	7.111769	8.700214	0.397	3.1265
NFI	≥ .95	0.932	0.962	0.941	0.922	0.999	0.990
TLI	≥ .95	0.914	0.955	0.917	0.895	1.006	0.978
CFI	≥ .95	0.942	0.971	0.948	0.930	1.000	0.993
RMSEA	< .05	0.116	0.084	0.123	0.138	0.000	0.072
	90% CI	0.097–0.136	0.063–0.105	0.100–0.147	0.116–0.161	0.000–0.112	0.010–0.140
SRMR	< .08	0.037	0.032	0.033	0.041	0.004	0.016
AIC		6988.509	6937.355	6047.702	6075.053	3743.130	3746.985
BIC		7009.278	6958.955	6065.979	6092.499	3753.930	3756.954

*Note. Df* degrees of freedom, *NFI* Normed fit Index, *TLI* Tucker Lewis Index, *CFI* Comparative Fit Index, *RMSEA* Root Mean Square Error of Approximation, *90% CI* RMSEA 90% Confidence Interval, *SRMR* Standardised Root Mean Square Residual, *AIC* Akaike Information Criterion, *BIC* Bayesian Information Criterion

### Distress level of the sample

To evaluate the level of distress, we divided the current sample into those scoring above and below 18.5 on the ADNM-8 and 8.5 on the ADNM-4. In a comparison of composite DASS-21 scores, an indicator of general psychological distress, the 303 individuals scoring above 18.5 on the ADNM-8 had significantly greater psychological distress (*M* = 20.43, *SD* = 10.68) than the 102 individuals scoring below (*M* = 9.87, *SD* = 7.80), mean difference = 10.56 (95% CI = 8.61, 12.50), *t*(237.26) = 10.70, *p* = 0.001, equal variances not assumed, 2000 bootstraps (as per [38]), *d* = 1.05,(95% CI = 0.82, 1.29) [39]. Similarly, 316 participants scored above 8.5 on the ADNM-4, and had significantly higher scores on the DASS-21 (*M* = 19.95, *SD* = 10.80) compared with participants below the cut point (*M* = 10.02, *SD* = 7.92), mean difference = 9.93 (95% CI = 7.89, 11.98), *t*(189.76) = 9.58, *p* = 001, equal variances not assumed, 2000 bootstraps, *d* = 0.97,(95% CI = 0.72, 1.12).

## Discussion

The results support the primary hypothesis of the study that the factor structure of the English versions of the ADNM-8 and ADNM-4 scales reflect the ICD-11 AjD symptom clusters for an Australian sample of adult cancer patients. Further, the findings are partially consistent with the remaining hypotheses and suggest that both the ADNM8 and ADNM-4 have good psychometric properties, and for cancer patients, their illness and the impact of COVID-19 were significant stressors. It is also clear that gender differences exist for stressors, with females reporting family conflicts and the death of a loved one significantly more often than men. In contrast, illness of a loved one and moving did not display gender differences as they have in earlier studies. Financial difficulty was the strongest predictor of AjD scores and not participants’ own serious illness as expected. However, these results do support own serious illness as the most straining stressor experienced by cancer patients. Finally, as expected, this study found the AjD scores of those with more or less recent diagnoses to be equivalent.

As predicted and consistent with earlier research, the results illustrate that AjD scales correlate positively with measures of psychological distress, depression, anxiety and stress [24, 31]. The current study found similar correlations between the ADNM-4 and measures of depression and anxiety as previously found [24]. Further, results indicated that the AjD scales negatively correlated with wellbeing. Negative correlations between the ADNM-8 and the WHO-5 were similar to those reported by Kazlauskas et al. [6], yet stronger than those reported by Ben-Ezra et al. [19]. In the current study, the ADNM-4 scale correlations with the wellbeing index, whilst in the same direction, were also stronger than those reported by Ben-Ezra et al. These findings lend support to the construct validity of the scales, are consistent with earlier studies, and are indicative of the high distress levels of the sample.

As expected, one’s own serious illness and the impact of COVID-19 were the most commonly reported stressors. Given the eligibility requirement of a cancer diagnosis to participate and the timing of the study’s recruitment occurring during the global pandemic, this result seems unremarkable. It was also expected there would be gender effects for the prevalence of stressors, with women reporting the death of a loved one, illness of a loved one, moving, and family conflicts more often than men. The study only found partial support for this forecast. The present results indicate that when compared with men, women did report the death of a loved one and family conflicts as stressors more frequently; however, they also reported the impact of COVID-19 more often, which was not anticipated. In a study of female breast cancer patients, Savard et al. [40] found that women reported, among other things*,* distress about attending treatment alone, social isolation, and family relationships. Given the immuno-vulnerability of cancer patients, and the isolation imposed by forced lockdowns, it seems natural that the impact of COVID-19 would constitute a serious stressor for many oncological patients.

The results did not support the hypothesis that a person's serious illness would be the strongest predictor of AjD. The strongest predictor was financial difficulties; Own serious illness was the second most influential predictor. Given the impact of the current pandemic on employment and the costs associated with cancer care, financial difficulties constitute a serious stressor for many oncological patients at this time. Newby et al. [41] found that about 50% of the participants in their study on mental health responses during COVID-19 reported moderate to extreme worry about their financial situation. Weissman et al. [42] found that financial worries were associated with serious psychological distress and that the odds of reporting two or more financial worries were higher for those who reported two or more chronic health conditions. Kale and Carroll [43] found in their study of 1380 cancer survivors from the United States, financial worries related to cancer were associated with a poorer quality of life, increased depressed mood, more frequent worry about their cancer returning and, *inter alia,* being female, being currently treated, being diagnosed between one and 3 years ago were significant predictors of subjective financial burden. Further, the average age of the current sample was 60, many of whom may no longer be earning a regular salary or, due to COVID-19 may be experienceing difficulties gaining sufficient work or regaining employment after a layoff, suggesting that financial worries are of particular importance for older people experiencing cancer.

Of interest was the variation in the strength of predictors for AjD symptom clusters. The preoccupation cluster saw family conflicts as the strongest predictor. It is possible that the forced isolation associated with lockdowns created significant worry and prevented resolution of any outstanding conflicts, exacerbated existing tensions, or created new ones, which led to rumination about family matters. In contrast, own serious illness and financial difficulties were the strongest predictors of the failure to adapt cluster of symptoms. Both predictors were 0.7 times stronger than the strongest predictor for the preoccupation subscale and were reported as having a significant impact on the daily lives of cancer patients interfering with the quality of life and their functional capacity. Previous research points to the likelihood that COVID-19 has a disproportionate impact on the economic status of cancer patients [44].

The results supported the hypothesis that cancer and its effects are reported as the most straining stressor experienced. However, it was frequently one's own serious illness within the context of COVID-19 that was referred to as the most straining stressor (e.g., “I have done nearly all my treatment (chemo and major surgery) without my family”). Such comments speak to the psychosocial impact of the COVID-19 pandemic on those living with cancer and are consistent with recent research [44, 45].

As predicted, the results suggest that the level of AjD is equivalent between those with a recent diagnosis and those with a diagnosis of longer than 12 months. This finding is consistent with findings by O’Donnell et al. [11] in their Australian study and lends support to their argument that not all AjD is transient when the consequence of a stressor is ongoing. The nature of cancer is pervasive in the lives of those with a diagnosis. The current sample included a broad range of times since diagnosis from 0.08 to 49 years, which speaks to the ongoing impact of cancer and its consequences on the lives of those who experience it. Cancer demands adjustment to a diagnosis and the treatment, its side effects, physical changes, living with pain, and the extensive impacts on one’s social, professional, and recreational life. Hence, cancer and other similar illnesses involve continual bouts of adaptation, which may lead to the chronicity of AjD, as suggested by O'Donnell et al.

Finally, the results support the prediction that the ADNM-8 and ADNM-4 would reflect the theoretical basis of the ICD-11 criteria and show a bifactor structure. The best fitting models in this research are the ADNM-8 Model 2 and ADNM-4 Model 5. These findings are consistent with the previous results by Kazlauskas et al. [6] though inconsistent with those of Ben-Ezra et al. [19] and Lavenda et al. [24] which used general population samples. In contrast, Kazlauskas used self-referred help-seekers for an AjD stress management program. This latter sample is more likely to be a distressed sample than those reflecting the general population and hence a closer sample to the current sample. The difference between the best fitting 8-item model from the Kazlauskas et al. [6] study and the study by Ben-Ezra et al. [19] may be understood by considering the exclusion of the functional impairment item in the latter study. Given the importance and relevance of functional impairment in oncological patients, this omission is significant. No real justification for excluding the functional impairment item in the ADNM-8 is provided [19]. Impairment in important life domains is a standard requirement for diagnosing most disorders in the DSM and ICD classification systems. As background to the preparation of the ICD-11, Evans et al.[46] investigated psychologists’ views about diagnostic classifications and found that over 80% of global respondents agreed that severity and functional impairment were important components of diagnosis. Impairment in critical areas of functioning is a requirement for diagnosing ICD-11 AjD and forms part of the failure to adapt symptom cluster. Clinically, functioning is a key indicator of a disorder’s severity. Functional impairment lies at the heart of a failure to adapt to new situations or demands. Omitting impairment from the scale may compromise the scale’s diagnostic utility. This may account for the similarity between the CFA results of the two ADNM-4 models tested in this study compared with the four ADNM-8 models examined given both exclude the functional impairment item.

Adjustment Disorder is a stress related disorder and cancer places individuals under a very specific and often sustained form of extreme stress. Screening for Adjustment Disorder allows clinicians to identify those at risk of developing the disorder (to rule out those who are not) and to intervene promptly by providing appropriate information and referral to available supports including psychologists especially those specializing in cancer and cancer pain, oncological social workers, as well as patient and carer support groups. Further the above services can be targeted to the presenting symptomology based on responses given in the questionnaires and the specific stressors identified. The aim of such supports may include counteracting maladaptive coping, reducing suicidality and general distress, improving cognitions and overall quality of life, reducing rumination, and developing active coping strategies for the patient and their family. Early intervention improves adherence to treatment and treatment outcomes [47]. As it is also suspected that AjD may develop into more severe psychopathology over time the early intervention through effective screening becomes about prevention of more psychologically debilitating conditions including those with increased risk of suicide and long-term influence on the person, their family, and their community.

### Limitations

The present study uses a cross-sectional design which limits drawing causal conclusions and generalising from the data. Central to this limitation is the fact that associations and predictions may exist in the opposite direction. Secondly, the generalisability of the findings is limited because the sample is not representative of the cancer patient population in that it was self-selected and has an overrepresentation of females. The study design did not include specific measures for more severe disorders (e.g. depression, posttraumatic stress disorder, or other stress related disorders) and therefore distinguishing between these diagnoses and adjustment disorder is not possible in the present study. A further point is that participation in the study was only available online.

### Future studies

Further investigation using longitudinal data would be useful in future research, as would the examination of AjD in cancer patients beyond the pandemic. Future research that establishes reliable cut off points for the current measures when used with cancer patients will improve the clinical utility of these screening instruments. Future research should include measures of more sever disorders (e.g., posttraumatic stress disorder, depression) in the design to allow differentiation between AjD and other disorders. A comparison of adjustment disorder in Australian non-oncological participants may strengthen the generalisability of the current findings and/or find differences worthy of future study. Further investigation of the impact of COVID-19 on rural and remote cancer sufferers and AjD would also be useful for future research as would examination of the differences between rural and urban instances of AjD in cancer patients and in a more representative Australian sample.

### Implications for practice

Based on these findings, it is reasonable to suggest that the ADNM-8 and ADNM-4 offer enhanced diagnostic usefulness in distinguishing those oncological patients at increased risk of AjD from those who are not. These scales offer a brief, reliable, valid assessment of AjD in oncological patients.

Given the strength of financial difficulties as a predictor of elevated levels of AjD, targeting interventions to assist individuals to better cope with financial stressors may be a useful clinical area to pursue. In addition, interventions for women that reduce rumination about COVID-19 and improve coping with family conflicts through skills development and improved thinking strategies may offer avenues to relieve cancer-associated stress and reduce preoccupation levels. In this sample, the predictiveness of own serious illness on failure to adapt was strong and may have implications for the target of interventions. Acceptance based interventions that develop increased psychological flexibility may be particularly valuable for those whose distress is more about difficulty adapting than preoccupation. Further, practical, and simple solutions for healthcare providers, such as supplying video call access for patients during COVID-19 cancer treatment, may help reduce the sense of isolation many cancer patients experience amidst the pandemic.

Oncologists may find it valuable to use these screeners one month post diagnosis to gauge the biopsychosocial risk for newly diagnosed cancer patients. As screeners these measures are not intended as diagnostic tools but as a means of identifying potential vulnerability and to rule out a disorder. The benefit of these brief screeners to oncologists is in the early detection of psychosocial vulnerability and subsequent risk of non-adherence to treatment, and poorer outcomes. These tools allow oncologists to identify psychosocial stressors and psychological distress secondary to cancer. Where such risks are identified the opportunity exists to intervene through prompt referral to appropriate services and supports. These screeners may also be used overtime to gauge the psychological trajectory from diagnosis to treatment and survivorship for each patient providing multiple opportunities for intervention and referral.

## Conclusion

The present study supports a two-factor structure of both brief ADNM scales consistent with the ICD-11 symptom clusters. The findings reinforce the ongoing nature of AjD in cancer patients, which lends support to the idea of AjD being chronic rather than transient in some contexts.

## Supplementary Information

**Additional file 1.** 

## Data Availability

The data that support the findings of this study are available from the corresponding author upon reasonable request.

## Abbreviations

ADNM-4: Adjustment Disorder New Module 4 -item
ADNM-8: Adjustment Disorder New Module 8 -item
AIC: Akaike Information Criterion
AjD: Adjustment disorder
APA: American Psychiatric Association
AIHW: Australian Institute of Health and Wellbeing
BIC: Bayesian Information Criterion
CFA: Confirmatory factor analysis
CFI: Comparative Fit Index
COVID-19: Coronavirus disease of 2019
DASS-21: Depression, Anxiety and Stress scale short form
df: Degrees of freedom
DSM-5: Diagnostic and Statistical Manual of Mental Disorders Fifth Edition
ICD-11: International Classification of Diseases 11^th^ Revision
NFI: Normed fit Index
RMSEA: Root Mean Square Error of Approximation
ROC: Receiver operating characteristic
SRMR: Standardised Root Mean Square Residual
TLI: Tucker Lewis Index
WHO: World Health Organization
WHO-5: World Health Organisation – Five Well-Being Index

## References

[1] Bray F, Ferlay J, Soerjomataram I, Siegel RL, Torre LA, Jemal A (2018). Global cancer statistics 2018: GLOBOCAN estimates of incidence and mortality worldwide for 36 cancers in 185 countries. Cancer J Clin.

[2] Australian Institute of Health and Welfare. (2021). Figure 2: Age-specific rates by sex and age group, 2021. All cancers combined in Cancer data in Australia: Cancer summary [Web Report]. Retrieved Jan 17, 2023, from https://www.aihw.gov.au/reports/cancer/cancer-data-in-australia/contents/cancer-summary-data-visualisation.

[3] Cancer Council NSW. Australian cancer prevalence exceeds 1 million: new estimates [press release]. 2016 Feb 2. https://www.cancercouncil.com.au/news/australian-cancer-prevalence-exceeds-1-million/.

[4] Bourke L, Boorjian SA, Briganti A, Klotz L, Mucci L, Resnick MJ (2015). Survivorship and improving quality of life in men with prostate cancer. Eur Urol.

[5] World Health Organization (2018). The ICD-11 classification of mental and behavioural disorders: Clinical descriptions and diagnostic guidelines.

[6] Kazlauskas E, Gegieckaite G, Eimontas J, Zelviene P, Maercker A (2018). A brief measure of the International Classification of Diseases-11 Adjustment Disorder: Investigation of psychometric properties in an adult help-seeking sample. Psychopathology.

[7] Hund B, Reuter K, Harter M, Brahler E, Faller H, Keller M (2016). Stressors, symptom profile, and predictors of adjustment disorder in cancer patients. Results from an epidemiological study with the Composite International Diagnostic Interview, adaptation for Oncology (CIDI-O). Depress Anxiety.

[8] Casey P, Jabbar F, O'Leary E, Doherty AM (2015). Suicidal behaviours in adjustment disorder and depressive episode. J Affect Disord.

[9] Gradus JL, Qin P, Lincoln AK, Miller M, Lawler E, Lash TL (2010). The association between adjustment disorder diagnosed at psychiatric treatment facilities and completed suicide. Clin Epidemiol.

[10] Kryzhanovskaya L, Canterbury R (2001). Suicidal behavior in patients with adjustment disorders. Crisis.

[11] O'Donnell ML, Alkemade N, Creamer M, McFarlane AC, Silove D, Bryant RA (2016). A longitudinal study of adjustment disorder after trauma exposure. Am J Psychiatry.

[12] Casey P, Bailey S (2011). Adjustment disorders: The state of the art. World Psychiatry.

[13] Abernathy BE (2009). The role of identity in posttraumatic growth and psychological adjustment for adults with cancer [Doctoral dissertation].

[14] Nock M, Borges G, Bromet E, Alonso J, Angermeyer M, Beautrais A (2008). Cross-national prevalence and risk factors for suicidal ideation, plans and attempts. Br J Psychiatry.

[15] Bachem R, Casey P (2018). Adjustment disorder: A diagnosis whose time has come. J Affect Disord.

[16] Mitchell AJ, Chan M, Bhatti H, Halton M, Grassi L, Johansen C (2011). Prevalence of depression, anxiety, and adjustment disorder in oncological, haematological, and palliative-care settings: a meta-analysis of 94 interview-based studies. Lancet Oncol.

[17] Horn AB, Boettcher VS, Holzer BM, Siebenhuener K, Maercker A, Battegay E (2019). Couples adjusting to multimorbidity: A dyadic study on disclosure and adjustment disorder symptoms. Front Psychol.

[18] Van Beek FE, Wijnhoven LMA, Custers JAE, Holtmaat K, De Rooij BH, Horevoorts NJE, et al. Adjustment disorder in cancer patients after treatment: prevalence and acceptance of psychological treatment. Support Care Cancer [Internet]. 2021;30(2):1797–1806. 10.1007/s00520-021-06530-0 PMC8486632 34599663

[19] Ben-Ezra M, Mahat-Shamir M, Lorenz L, Lavenda O, Maercker A (2018). Screening of adjustment disorder: Scale based on the ICD-11 and the Adjustment Disorder New Module. J Psychiatr Res.

[20] Maercker A, Brewin CR, Bryant RA, Cloitre M, Van Ommeren M, Jones LM (2013). Diagnosis and classification of disorders specifically associated with stress: proposals for ICD-11. World Psychiatry.

[21] Einsle F, Kollner V, Dannemann S, Maercker A (2010). Development and validation of a self-report for the assessment of adjustment disorders. Psychol Health Med.

[22] Shevlin M, Hyland P, Ben-Ezra M, Karatzias T, Cloitre M, Vallieres F (2020). Measuring ICD-11 adjustment disorder: the development and initial validation of the International Adjustment Disorder Questionnaire. Acta Psychiatr Scand.

[23] O'Donnell ML, Agathos J, Metcalf O, Gibson K, Lau W (2019). Adjustment disorder: Current developments and future directions. Int J Environ Res Public Health.

[24] Lavenda O, Mahat-Shamir M, Lorenz L, Hamama-Raz Y, Greenblatt-Kimron L, Pitcho-Prelorentzos S (2019). Revalidation of Adjustment Disorder-New Module-4 screening of adjustment disorder in a non-clinical sample: Psychometric reevaluation and correlates with other ICD-11 mental disorders. PsyCh Journal.

[25] World Health Organization. WHO (Five) Well-Being Index (English version). Geneva 1998. https://www.psykiatri-regionh.dk/who-5/Pages/default.aspx.

[26] Topp CW, Ostergaard SD, Sondergaard S, Bech P (2015). The WHO-5 Well-Being Index: A systematic review of the literature. Psychother Psychosom.

[27] Hoffman CJ, Ersser SJ, Hopkinson JB, Nicholls PG, Harrington JE, Thomas PW (2012). Effectiveness of mindfulness-based stress reduction in mood, breast- and endocrine-related quality of life, and well-being in stage 0 to III breast cancer: A randomized, controlled trial. J Clin Oncol.

[28] Lovibond SH, Lovibond PF (1995). Manual for the Depression Anxiety Stress Scales.

[29] Henry JD, Crawford JR (2005). The short-form version of the Depression Anxiety Stress Scales (DASS-21): Construct validity and normative data in a large non-clinical sample. Br J Clin Psychol.

[30] Antony MM, Bieling PJ, Cox BJ, Enns MW, Swinson RP (1998). Psychometric properties of the 42-item and 21-item versions of the Depression Anxiety Stress Scales in clinical groups and a community sample. Psychol Assess.

[31] Lorenz L, Bachem RC, Maercker A (2016). The Adjustment Disorder-New Module 20 as a Screening Instrument: Cluster Analysis and Cut-off Values. The Int J Occup Environ Med.

[32] Fox RS, Lillis T, Gerhart J, Hoerger M, Duberstein P (2018). Multiple group confirmatory factor analysis (CFA) of the DASS-21 Depression and Anxiety Scales: How do they perform in a cancer sample?. Psychol Rep.

[33] Crawford JR, Cayley C, Lovibond PF, Wilson PH, Hartley C (2020). Percentile Norms and Accompanying Interval Estimates from an Australian General Adult Population Sample for Self-Report Mood Scales (BAI, BDI, CRSD, CES-D, DASS, DASS-21, STAI-X, STAI-Y, SRDS, and SRAS). Aust Psychol.

[34] Soper D (2022). Structural Equation Model Sample Size Calculator.

[35] Tabachnick, B. G., Fidell, L. S. Using Multivariate Statistics: Pearson New International Edition, 6th Edition. Pearson (Intl); 20130827. Retrieved from vbk://978129203454620130827.

[36] Medsker GJ, Williams LJ, Holahan PJ (1994). A review of current practices for evaluating causal models in organizational behavior and human resources management research. J Manag.

[37] Gignac GE, Szodorai ET (2016). Effect size guidelines for individual differences researchers. Pers Individ Dif.

[38] Pek J, Wong O, Wong ACM (2018). How to Address Non-normality: A Taxonomy of Approaches, Reviewed, and Illustrated. Front Psychol.

[39] Pek J, Flora DB (2018). Reporting effect sizes in original psychological research: A discussion and tutorial. Psychol Methods.

[40] Savard J, Jobin-Theberge A, Massicotte V, Banville C (2021). How did women with breast cancer experience the first wave of the COVID-19 pandemic? A qualitative study.. Support Care Cancer.

[41] Newby JM, O'Moore K, Tang S, Christensen H, Faasse K (2020). Acute mental health responses during the COVID-19 pandemic in Australia. PLoS ONE.

[42] Weissman J, Russell D, Mann JJ (2020). Sociodemographic Characteristics, Financial Worries and Serious Psychological Distress in U.S. Adults. Community Ment Health J.

[43] Kale HP, Carroll NV (2016). Self-reported financial burden of cancer care and its effect on physical and mental health-related quality of life among US cancer survivors. Cancer.

[44] Jammu AS, Chasen MR, Lofters AK, Bhargava R (2021). Systematic rapid living review of the impact of the COVID-19 pandemic on cancer survivors: update to August 27, 2020. Support Care Cancer.

[45] Romito F, Dellino M, Loseto G, Opinto G, Silvestris E, Cormio C (2020). Psychological Distress in Outpatients With Lymphoma During the COVID-19 Pandemic. Front Oncol.

[46] Evans SC, Reed GM, Roberts MC, Esparza P, Watts AD, Correia JM (2013). Psychologists' perspectives on the diagnostic classification of mental disorders: results from the WHO-IUPsyS Global Survey. Int J Psychol.

[47] Grassi L, Caruso R, Sabato S, Massarenti S, Nanni MG, The UniFe Psychiatry Working Group C (2014). Psychosocial screening and assessment in oncology and palliative care settings. Front Psychol.

