# Supplementary material for: Validation of the brief Adjustment Disorder New Modules with Australian oncology patients
Source: Biopsychosoc Med. 2023 Jan 25;17:2. doi: 10.1186/s13030-022-00259-w (PMC9875190; doi:10.1186/s13030-022-00259-w)
Supplement: Supplementary file 1 — Additional file 1. [file 13030_2022_259_MOESM1_ESM.docx]

**Validation of the Brief Adjustment Disorder New Modules with Australian Oncology Patients**

# Supplementary Material

Multiple regression analysis examined the predictiveness of stressors. Due to the broad range in the time since diagnosis data (from 0.08 to 49 years), secondary analyses were conducted to consider if the length of time since diagnosis influenced the stressors reported. Statistical analysis was completed to examine the stressor profiles for those with a diagnosis of less than one year, five years, seven and a half years and ten years. These time points were selected based on the positive skew of the sample (3.00). The details of each analysis follow. For those participants whose diagnosis was one year or less, the nineteen stressors accounted for 35% of the variance in ADNM-8 AjD scores, *R*^2^ = .35, *F*(19.98) = 2.77, *p* = .001. This split reflects 29% of cases in the data set. The regression results appear in Table 1.

Table 1

Unstandardised (B) and Standardised (β) Regression Coefficients and Semi-Partial Correlations (sr) for Life Stressors as Predictors of ADNM-8 AjD Scores for those with a cancer diagnosis of 1 year or less (N = 118)

| Life stressors | *B* | 95% CI for *B* | | β | *t* | *p* | | *sr* |
| --- | --- | --- | --- | --- | --- | --- | --- | --- |
|  |  | *LL* | *UL* |  |  |  |  |  |
| The impact of COVID-19 | 3.05 | 1.02 | 5.09 | .28 | 2.97 | .004 | | .24 |
| Acute stressors |  |  |  |  |  |  | |  |
| Death of a loved one | -2.69 | -4.77 | -0.60 | -.23 | -2.56 | .012 | | -.21 |
| Divorce/separation | 1.20 | -3.03 | 5.42 | .05 | 0.56 | .575 | | .05 |
| Moving | -.83 | -3.33 | 1.67 | -.07 | -0.66 | .512 | | -.05 |
| Assault / Criminal act | -.66 | -12.25 | 10.93 | -.01 | -.11 | .910 | | -.01 |
| Retirement | -.36 | -2.71 | 1.99 | -.03 | -.30 | .762 | | -.03 |
| Termination of an important leisure activity | 1.07 | -1.54 | 3.68 | .08 | .81 | .418 | | .07 |
| Serious accident | 1.43 | -4.08 | 6.94 | .05 | .52 | .607 | | .04 |
| Natural disasters | 9.57 | 3.08 | 16.05 | .31 | 2.93 | .004 | | .24 |
| Chronic stressors |  |  |  |  |  |  | |  |
| Financial difficulties | 2.51 | .58 | 4.44 | .25 | 2.58 | .011 | | .21 |
| Family conflict | 1.78 | .00 | 3.56 | .17 | 1.98 | .050 | | .16 |
| Own serious illness | .53 | -1.73 | 2.79 | .05 | .47 | .642 | | .04 |
| Conflict at work | .03 | -2.29 | 2.35 | .00 | .02 | .981 | | .00 |
| Conflict with neighbours | .50 | -2.83 | 3.84 | .03 | .30 | .769 | | .02 |
| Too much/ Too little work | .73 | -1.37 | 2.83 | .07 | .69 | .494 | | .06 |
| Illness/care of a loved one | -.28 | -2.06 | 1.51 | -.03 | -.31 | .759 | | -.03 |
| Unemployment | 1.01 | -1.60 | 3.63 | .08 | .77 | .445 | | .06 |
| Pressure to meet deadlines | -.52 | -2.98 | 1.95 | -.04 | -.42 | .679 | | -.03 |
| Other | 2.16 | -2.7 | 6.39 | .09 | 1.01 | .313 | | .08 |
| *Note*. CI = Confidence interval. *LL* = lower limit, *UL* = upper limit. | | | | | | |  | |

For those participants whose diagnosis was five years or less, the nineteen stressors accounted for 28% of the variance in ADNM-8 AjD scores, *R*^2^ = .28, *F*(19, 276) = 5.62, *p* = .001. This split reflects 73% of cases in the data set. The regression results appear in Table 2.

Table 2

Unstandardised (B) and Standardised (β) Regression Coefficients and Semi-Partial Correlations (sr) for Life Stressors as Predictors of ADNM-8 AjD Scores for those with a cancer diagnosis of 5 years or less (N = 296)

| Life stressors | *B* | 95% CI for *B* | | β | *t* | *p* | | *sr* |
| --- | --- | --- | --- | --- | --- | --- | --- | --- |
|  |  | *LL* | *UL* |  |  |  |  |  |
| The impact of COVID-19 | 1.5 | .29 | 2.72 | .14 | 2.43 | .016 | | .12 |
| Acute stressors |  |  |  |  |  |  | |  |
| Death of a loved one | -1.57 | -2.91 | -.23 | -.12 | -2.31 | .022 | | -.12 |
| Divorce/separation | 1.82 | -.78 | 4.42 | .08 | 1.38 | .170 | | .07 |
| Moving | -.31 | -1.99 | 1.37 | -.02 | -.36 | .72 | | -.02 |
| Assault / Criminal act | .52 | -4.12 | 5.15 | .01 | .22 | .827 | | .01 |
| Retirement | -.10 | -1.57 | 1.37 | -.01 | -.13 | .894 | | -.01 |
| Termination of an important leisure activity | -.01 | -1.53 | 1.51 | .00 | -.01 | .991 | | -.00 |
| Serious accident | 1.37 | -2.35 | 5.08 | .04 | .73 | .469 | | .04 |
| Natural disasters | 9.11 | 3.28 | 14.94 | .17 | 3.08 | .002 | | .16 |
| Chronic stressors |  |  |  |  |  |  | |  |
| Financial difficulties | 2.71 | 1.44 | 3.99 | .23 | 4.19 | .001 | | .21 |
| Family conflict | 2.28 | 1.08 | 3.49 | .20 | 3.72 | .001 | | .19 |
| Own serious illness | 1.99 | .70 | 3.29 | .17 | 3.04 | .003 | | .16 |
| Conflict at work | 1.24 | -.28 | 2.76 | .09 | 1.61 | .108 | | .08 |
| Conflict with neighbours | 2.22 | .17 | 4.27 | .12 | 2.13 | .03 | | .11 |
| Too much/ Too little work | .96 | -.44 | 2.37 | .08 | 1.35 | .179 | | .07 |
| Illness/care of a loved one | .44 | -.79 | 1.66 | .04 | .70 | .482 | | .04 |
| Unemployment | .09 | -1.58 | 1.76 | .01 | .11 | .916 | | .01 |
| Pressure to meet deadlines | -.43 | -2.08 | 1.22 | -.03 | -.51 | .611 | | -.03 |
| Other | 2.66 | .03 | 5.29 | .11 | 1.99 | .048 | | .10 |
| *Note*. CI = Confidence interval. *LL* = lower limit, *UL* = upper limit. | | | | | | |  | |

For those participants whose diagnosis was seven and a half years or less, the nineteen stressors accounted for 26% of the variance in ADNM-8 AjD scores, *R*^2^ = .26, *F*(19.308) = 5.82, *p* = .001. This split reflects 81% of cases in the data set. The regression results appear in Table 3.

Table 3

Unstandardised (B) and Standardised (β) Regression Coefficients and Semi-Partial Correlations (sr) for Life Stressors as Predictors of ADNM-8 AjD Scores for those with a cancer diagnosis of 7.5 years or less (N = 328)

| Life stressors | *B* | 95% CI for *B* | | β | *t* | *p* | | *sr* |
| --- | --- | --- | --- | --- | --- | --- | --- | --- |
|  |  | *LL* | *UL* |  |  |  |  |  |
| The impact of COVID-19 | 1.66 | .52 | 2.80 | .15 | 2.86 | .005 | | .14 |
| Acute stressors |  |  |  |  |  |  | |  |
| Death of a loved one | -1.32 | -2.61 | -.03 | -.10 | -2.01 | .046 | | -.10 |
| Divorce/separation | 2.28 | -.17 | 4.73 | .09 | 1.83 | .068 | | .09 |
| Moving | -.17 | -1.74 | 1.40 | -.01 | -.21 | .833 | | -.01 |
| Assault / Criminal act | .51 | -4.15 | 5.17 | .01 | .22 | .830 | | .01 |
| Retirement | .23 | -1.19 | 1.66 | .02 | .32 | .747 | | .02 |
| Termination of an important leisure activity | .04 | -1.46 | 1.53 | .00 | .05 | .963 | | .00 |
| Serious accident | .42 | -2.87 | 3.72 | .01 | .25 | .80 | | .01 |
| Natural disasters | 8.70 | 2.82 | 14.57 | .15 | 2.91 | .004 | | .14 |
| Chronic stressors |  |  |  |  |  |  | |  |
| Financial difficulties | 2.31 | 1.08 | 3.54 | .20 | 3.70 | .001 | | .18 |
| Family conflict | 2.12 | .97 | 3.28 | .19 | 3.61 | .001 | | .18 |
| Own serious illness | 1.81 | .63 | 2.99 | .15 | 3.02 | .003 | | .15 |
| Conflict at work | 1.44 | -.04 | 2.92 | .10 | 1.91 | .057 | | .09 |
| Conflict with neighbours | 2.44 | .51 | 4.36 | .13 | 2.49 | .013 | | .12 |
| Too much/ Too little work | .71 | -.65 | 2.07 | .06 | 1.02 | .307 | | .05 |
| Illness/care of a loved one | .48 | -.69 | 1.65 | .04 | .81 | .421 | | .04 |
| Unemployment | .57 | -1.05 | 2.19 | .04 | .69 | .489 | | .03 |
| Pressure to meet deadlines | -.15 | -1.74 | 1.45 | -.01 | -.18 | .857 | | -.01 |
| Other | 2.57 | .10 | 5.05 | .10 | 2.05 | .042 | | .10 |
| *Note*. CI = Confidence interval. *LL* = lower limit, *UL* = upper limit. | | | | | | |  | |

For those participants whose diagnosis was ten years or less, the nineteen stressors accounted for 29 % of the variance in ADNM-8 AjD scores, *R*^2^ = .29, *F*(19.331) = 7.09, *p* = .001. This split reflects 87% of cases in the data set. The regression results appear in Table 4.

Table 4

Unstandardised (B) and Standardised (β) Regression Coefficients and Semi-Partial Correlations (sr) for Life Stressors as Predictors of ADNM-8 AjD Scores for those with a cancer diagnosis of 10 years or less (N = 351)

| Life stressors | *B* | 95% CI for *B* | | β | *t* | *p* | | *sr* |
| --- | --- | --- | --- | --- | --- | --- | --- | --- |
|  |  | *LL* | *UL* |  |  |  |  |  |
| The impact of COVID-19 | 1.52 | .42 | 2.62 | .13 | 2.71 | .007 | | .13 |
| Acute stressors |  |  |  |  |  |  | |  |
| Death of a loved one | -1.20 | -2.46 | .07 | -.09 | -1.85 | .065 | | -.09 |
| Divorce/separation | 2.34 | -.06 | 4.73 | .09 | 1.92 | .056 | | .09 |
| Moving | -.18 | -1.74 | 1.37 | -.01 | -.23 | .818 | | -.01 |
| Assault / Criminal act | .55 | -4.07 | 5.18 | .01 | .24 | .814 | | .01 |
| Retirement | .21 | -1.14 | 1.57 | .02 | .31 | .757 | | .01 |
| Termination of an important leisure activity | -.19 | -1.61 | 1.24 | -.01 | -.26 | .793 | | -.01 |
| Serious accident | .83 | -2.30 | 3.97 | .03 | .52 | .601 | | .02 |
| Natural disasters | 7.09 | 2.05 | 12.13 | .13 | 2.77 | .006 | | .13 |
| Chronic stressors |  |  |  |  |  |  | |  |
| Financial difficulties | 2.38 | 1.19 | 3.57 | .20 | 3.93 | .001 | | .18 |
| Family conflict | 2.28 | 1.16 | 3.40 | .19 | 4.00 | .001 | | .19 |
| Own serious illness | 2.34 | 1.22 | 3.46 | .20 | 4.10 | .001 | | .19 |
| Conflict at work | 1.29 | -.12 | 2.70 | .09 | 1.81 | .072 | | .08 |
| Conflict with neighbours | 2.64 | .72 | 4.56 | .13 | 2.70 | .007 | | .13 |
| Too much/ Too little work | .67 | -.65 | 1.99 | .05 | 1.00 | .320 | | .05 |
| Illness/care of a loved one | .78 | -.35 | 1.92 | .07 | 1.35 | .177 | | .06 |
| Unemployment | .76 | -.84 | 2.36 | .05 | .93 | .353 | | .04 |
| Pressure to meet deadlines | .12 | -1.42 | 1.67 | .01 | .16 | .874 | | .01 |
| Other | 3.06 | .78 | 5.34 | .13 | 2.64 | .009 | | .12 |
| *Note*. CI = Confidence interval. *LL* = lower limit, *UL* = upper limit. | | | | | | |  | |
